# Strategies for Preventing and Treating Oral Mucosal Infections Associated with Removable Dentures: A Scoping Review

**DOI:** 10.3390/antibiotics13030273

**Published:** 2024-03-18

**Authors:** Adriana Barbosa Ribeiro, Pillar Gonçalves Pizziolo, Lorena Mosconi Clemente, Helena Cristina Aguiar, Beatriz de Camargo Poker, Arthur Augusto Martins e Silva, Laís Ranieri Makrakis, Marco Aurelio Fifolato, Giulia Cristina Souza, Viviane de Cássia Oliveira, Evandro Watanabe, Cláudia Helena Lovato da Silva

**Affiliations:** 1 Department of Dental Materials and Prosthesis, Ribeirão Preto School of Dentistry, University of São Paulo, Café Avenue S/N, Ribeirão Preto 14040-904, SP, Brazil; driribeiro@usp.br (A.B.R.); pillarpizziolo@usp.br (P.G.P.); lorena.clemente@usp.br (L.M.C.); hcaguiar@usp.br (H.C.A.); beatrizpoker@usp.br (B.d.C.P.); arthur.amsilva@usp.br (A.A.M.e.S.); lais.makrakis@usp.br (L.R.M.); marcofifolato@usp.br (M.A.F.); giucsouza99@usp.br (G.C.S.); vivianecassia@usp.br (V.d.C.O.); 2 Department of Restorative Dentistry, Ribeirão Preto School of Dentistry, University of São Paulo, Café Avenue S/N, Ribeirão Preto 14040-904, SP, Brazil; ewatanabe@forp.usp.br

**Keywords:** biofilms, infection control, antifungal agents, *Candida albicans*, stomatitis, denture, oral hygiene, denture cleansers, denture, complete, acrylic resins, review

## Abstract

Oral infections occur due to contact between biofilm rich in *Candida albicans* formed on the inner surface of complete dentures and the mucosa. This study investigated historical advances in the prevention and treatment of oral mucosal infection and identified gaps in the literature. Bibliographic research was conducted, looking at PubMed, Embase, Web of Science, and Scopus, where 935 articles were found. After removing duplicates and excluding articles by reading the title and abstract, 131 articles were selected for full reading and 104 articles were included. Another 38 articles were added from the gray literature. This review followed the PRISMA-ScR guidelines. The historical period described ranges from 1969 to 2023, in which, during the 21st century, in vitro and in vivo studies became more common and, from 2010 to 2023, the number of randomized controlled trials increased. Among the various approaches tested are the incorporation of antimicrobial products into prosthetic materials, the improvement of oral and denture hygiene protocols, the development of synthetic and natural products for the chemical control of microorganisms, and intervention with local or systemic antimicrobial agents. Studies report good results with brushing combined with sodium hypochlorite, and new disinfectant solutions and products incorporated into prosthetic materials are promising.

## 1. Introduction

Dental prosthesis aims to restore some or all missing teeth, as well as their adjacent structures, improving aesthetics, function, and patient’s quality of life [1]. Conventional complete and partial removable dentures are widely used due to their affordability and fulfill a rehabilitative role in the stomatognathic system. However, care and hygiene practices are necessary for the individuals’ maintenance of their oral and general health [2,3,4,5].

Denture manufacturing materials are prone to biofilm formation, favoring the development of inflammation when in direct contact with the support mucosa [4,6,7,8,9,10,11,12,13,14,15]. Upper complete dentures are commonly associated with subjacent mucosa inflammation [9,16], although the inner surface of lower complete dentures is also a biofilm reservoir [8]. This biofilm is composed of a complex structure of mucins, proteins, and polymucosaccharides, similar to dental biofilm, except for the increased quantity of *Candida* spp., especially *Candida albicans* [17,18,19,20,21]. One of the most prevalent inflammations in removable denture users is denture stomatitis. This chronic disease affects the mucosa adjacent to the removable dentures and, despite its multifactorial etiology [21], studies indicate that the microbial load of *Candida* spp., especially *C. albicans*, is the main etiological factor [2,18,21].

Denture hygiene can be achieved using mechanical and chemical methods [8,22]. Among the mechanical methods, brushing, microwaves, and ultrasound may be recommended. Brushing is the most used [23,24] and can be performed with brushes associated with common toothpastes, natural [19,25,26,27] or synthetic antimicrobial agents [28], or soap [29]. When denture brushing is associated with mucosa brushing, the level of inflammation seems to decrease [16,30,31,32,33]. Regarding the limitations of current hygiene practices, a cross-sectional study revealed a significant correlation between the frequency of denture cleaning practices and educational levels. As the level of education increased, a statistically significant increase in adherence to denture hygiene routines was observed. Furthermore, the research suggests that the prevalent lack of adequate hygiene practices is attributable to a variety of factors, including diverse oral hygiene habits, physical ability, manual dexterity, motivation, awareness, educational background, and the availability of supervision [14].

Additionally, the literature suggests that the ideal hygiene method is to associate cleaning solutions for immersing dentures with brushing. The most used solutions are alkaline peroxides [18,19,23,24,34,35,36,37,38,39,40,41,42,43,44,45,46,47,48,49], sodium hypochlorite [2,19,41,44,49,50], and chlorhexidine [19,45,46,50,51,52]. Regarding the immersion time, subjects usually keep their dentures immersed in these solutions overnight [18] and the literature shows that long immersion periods impact tissue inflammation reduction [40,49]; however, they can cause adverse effects on the materials used in the manufacturing of dentures [53]. Furthermore, alternative methods for controlling biofilm, such as altering the surface of the acrylic resin through glaze application [53,54,55,56,57,58], the use of ozone [59], adhesives with chitosan [60], and henna [61] can be found in the literature.

In addition to denture hygiene methods for treatment of inflammation and/or mucosal infections, the literature indicates the use of a low-power laser [62], antifungals for local or systemic application, whether associated or not with photodynamic therapy [50,52,63,64,65,66,67,68,69,70,71,72], combined treatments using drugs with a local effect for stomatitis [73] and drugs with a systemic effect for patients with gastrointestinal pathologies, type 2 diabetes [74], or pathologies in the cardiovascular system [75]. Although less used, products based on organic acid, titanium dioxide, and inorganic silver antibacterial agents, and chlorine dioxide, as well as natural products used for immersion or incorporation into prosthetic materials, are also found in the literature [7,19,76,77,78,79,80,81,82,83,84,85,86,87,88,89,90].

Consulting the literature, it appears that the interest in studying biofilm control methods and preventing oral infections is long-standing and vast, given the large number of published clinical and in vitro studies. However, the research indicates that hygiene is still poor among denture wearers due to the limited knowledge of denture cleaning and oral hygiene practices by the majority of the denture wearers [91]. Therefore, emphasizing the importance of periodic consultations on the evaluation and maintenance of prostheses, as well as reinforcing guidance on hygiene methods, is essential for maintaining good general health and increasing adherence to adequate oral hygiene practices [91].

Furthermore, it is essential that professionals find information, with scientific evidence, about the most appropriate methods for preventing and treating oral mucosal infections, which will contribute to their providing consistent and accurate information to patients. To help with this, the current literature review aims to present a brief contextualization of inflammations of the oral mucosa and a historical report of in vitro and clinical studies on methods of preventing and treating infections in the oral mucosa related to the use of removable dentures, and to identify any gaps that still need to be answered related to this topic.

## 2. Results

### 2.1. Search Results

**Figure 1:**
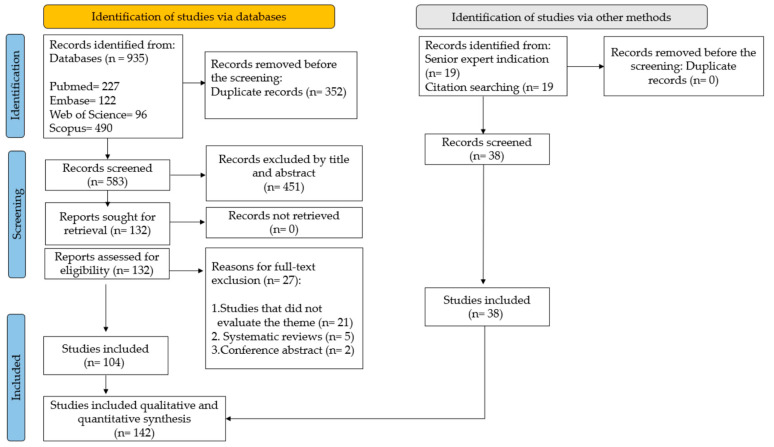
Study workflow.

The literature search retrieved 935 articles: 227 from PubMed, 122 from Embase, 96 from Web of Science, and 490 from Scopus. After the removal of duplicates (n = 352), 583 articles remained. The by-title/abstract screening resulted in the exclusion of 452 articles. The full text of the remaining 131 articles was screened, and 104 of them were selected [2,3,4,5,6,7,8,9,10,11,12,13,14,15,16,18,22,23,24,25,26,27,28,29,30,31,32,33,34,35,36,37,40,41,43,44,45,47,49,50,51,52,53,54,55,56,57,58,59,60,61,62,63,64,65,66,67,68,69,70,71,72,73,74,75,76,77,78,79,80,81,82,83,84,85,86,87,88,90,92,93,94,95,96,97,98,99,100,101,102,103,104,105,106,107,108,109,110,111,112,113,114,115,116]. Another 38 articles were found in the gray literature, 19 by a senior expert indication [19,39,89,117,118,119,120,121,122,123,124,125,126,127,128,129,130,131,132], and 19 from searching citations [38,48,133,134,135,136,137,138,139,140,141,142,143,144,145,146,147,148,149], resulting in a total of 142 articles for the scoping review, according to the study workflow in Figure 1. Another 21 articles were used to describe the essential definitions in Section 2.2.1 and Section 3 [1,17,20,21,42,46,91,150,151,152,153,154,155,156,157,158,159,160,161,162,163].

### 2.2. Literature Review

#### 2.2.1. Main Inflammations/Infections Related to the Use of removable Prostheses: Etiology and Diagnosis

The spectrum of denture-related mucosal lesions includes traumatic ulcer, denture-related stomatitis (DRS), angular cheilitis, and combinations of these lesions [9,70,150,151,152]. Traumatic ulcers are commonly caused by maladaptation of the denture, while DRS and angular cheilitis occur due to infections caused by *Candida* and loss of vertical dimension, respectively. Angular cheilitis can be aggravated by *Candida* infection.

*Candida* is a type of fungi in the form of yeast [9,70,152], which is part of the normal microbiota of completely edentulous patients and interacts with bacteria such as *Aggregatibacter actinomycetemcomitans*, *Fusobacterium nucleatum*, *Eikenella corrodens*, *Capnocytophaga* spp., *Campylobacter concisus*, *Streptococcus mitis*, *Streptococcus gordonii*, *Streptococcus constellatus*, *Streptococcus mutans*, *Staphylococcus* spp., *Neisseria* spp., *Actinomyces odontolyticus*, and *Veillonella parvula*, and is also commonly isolated in complete denture biofilm [24,40,49,92].

DRS usually occurs beneath denture-bearing areas and is the most common infection in elderly individuals who are complete denture users [9,14,16,150,153,154]. Classically, DRS is characterized by erythema and edema of the mucosa, dysgeusia, and a burning sensation. However, a recent study showed that there was no significant difference among groups with or without DRS relating to complaints of burning mouth or dysgeusia [21], which makes this disease not noticeable to the patient.

The literature shows that the risk factors of denture stomatitis are advanced age, altered health conditions, the use of complete dentures when compared to the use of removable partial dentures, poor denture fit, poor denture hygiene, and colonization by *C. albicans* on the surface of the denture and oral mucosa [14,155]. However, the microbial load of *Candida* spp. is the most relevant causal factor of DRS, leading to the capacity for local signaling through IL-6 [21]. The *C. albicans* biofilm on dentures is heterogeneous and thick, which makes the mechanical and antimicrobial action of hygiene procedures difficult [41,49,73].

Concerning the diagnosis of DRS, the accuracy depends on the educational background and the experience of the researcher or clinicians. In some cases, collaboration with a physician may be necessary to uncover any underlying immunocompromising conditions, ensuring an accurate diagnosis and effective treatment [152]. As an auxiliary method for the diagnosis of the DRS, the professional can use clinical indices associated with culture techniques via the identification and quantification of *Candida* spp. [156].

Newton’s classification was the first clinical indices proposed by the diagnostics of DRS [157], which classify the DRS into three levels: Type I (pinpoint hyperemia—localized or sparse palatal erythema), Type II (diffuse hyperemia—diffuse erythema, more common), and Type III (granular hyperemia—papillary hyperplasia with rough or nodular mucosa).

Over time, other indices were proposed to evaluate the inflammation associated with the erythema scale based on Newton’s classification and the presence of the *Candida* [34]. In 2014, assessments of DRS using a total inflammation score were proposed based on the area and intensity of inflammation (range: 0–6), using a modified version of Newton’s classification, in addition to the inflammation area index and inflammation severity index. For this, the authors used a modification of the Newton’s classification (0: healthy mucosa; Type IA: petechiae in normal palatal tissue, usually found around the orifices of the ducts of the palatal mucous glands; Type IB: localized area of inflammation of the denture-bearing area; Type II: generalized area of inflammation of the denture-bearing area; Type III: hyperplastic palatal surface with inflammation of the denture) plus the inflammation area index (0: no inflammation; 1: inflammation of the palate extending up to 25% of the palatal, denture-bearing tissue; 2: inflammation of the palate covering between 25% and 50% of the palatal denture-bearing tissue; 3: inflammation covering more than 50% of the palatal denture-bearing tissue), and the inflammation severity index (0: normal tissue; 1: mild inflammation—slight redness, no swelling or edema; 2: moderate inflammation—redness with some edema; 3: severe inflammation—acutely inflamed redness, edema) [32]. Another proposal was presented in which the clinical severity of DRS was evaluated considering the extension of the type of hyperemia on the area covered by the denture and affected by palatal inflammation, as well as the degree of erythema. The severity classification of the DRS ranges from score 1, which represents a Newton Type I (punctiform hyperemia), affecting one quadrant (regardless of which) with a less intense degree of erythema, up to score 24, which refers to a Type III, covering the four quadrants with increased redness [158]. However, by our acknowledgment, Newton’s Classification is the most used method, followed by Newton’s Classification modified [32].

#### 2.2.2. Prevention and Treatment of Oral Diseases Related to the Use of Dentures

The literature contains several studies with proposals for the prevention and treatment of inflammations/infections. These studies can be grouped into three large groups, namely:

1. Studies for topical and systemic treatments using antimicrobial agents, either alone or associated with local interventions (Table 1);
2. Studies for prevention and local treatment using mechanical, chemical, physical, and associated hygiene methods (Table 2);
3. Studies for prevention and local treatment using material modifications (Table 3).

Between 1969 and 1989, various treatments involving antifungals such as nystatin, coupled with the relining of prostheses and subsequent replacement, were proposed [93]. Amphotericin, administered in 10 mg tablets [52] or in the form of 2% patches [76], was also considered during this period. These therapeutic approaches demonstrated temporary efficacy in reducing inflammation and microbial fungal load. However, oral microflora was observed to reestablish itself after two weeks.

Notably, the combination of antifungals with the surgical removal of hyperplastic tissue in advanced cases of DRS, followed by the relining of prostheses until the installation of new prostheses, proved to be efficient [64]. The association of prosthesis relining with the surgical procedure offers a treatment characterized by a brief healing period, the absence of pain and bleeding, and a low incidence of post-surgical complications [63,94].

Methods aimed at treating and preventing inflammation through the mechanical action of brushing the prosthesis and mucosa have been documented and have proven effective in diminishing inflammatory severity [30,31]. However, the efficacy of this approach hinges on the dentist’s commitment to patient education, fostering an understanding of biofilm visualization, and ensuring proficient brushing techniques [2,31].

During the same timeframe, the utilization of chlorhexidine digluconate as a disinfectant, available in the form of 2% or 0.2% solutions [36,52], 1% gels [95], or tablets of 5 mg [52], was assessed, revealing positive outcomes in terms of DRS improvement. However, a relapse was observed within two weeks following the cessation of treatment.

In an endeavor to deter biofilm formation on complete dentures and address DRS, a clinical study [54] was conducted to assess the application of a glazing polyfunctional acrylic monomer (Perma Link, G.C. Internat. Cooperation Tokyo, Japan). This was carried out in conjunction with a photopolymerizing diluent, a photopolymerizing initiator, and an ultraviolet light curing apparatus (Perma Cure UC-1 10) applied to the internal surface of the prosthesis. The alteration of the prosthesis surface contributed to a notable reduction in erythema and diminished biofilm accumulation on the prosthesis.

Over time, and in response to the evolving needs of the edentulous population and denture users, it is discernible that the literature has progressed with the necessity to clarify the evidence. Notably, between 1990 and 2009, there was a marked escalation in the production of both in vitro and clinical studies.

Several studies conducted during this period focused their objectives on evaluating various antifungal agents. These included amphotericin B tablets [52], natural antifungals derived from salivary polypeptides rich in histidine [65], the topical application of miconazole 2% in gel, or systemic treatment with fluconazole 50 mg [96]. Additionally, another study investigated the topical application of miconazole (55 mg/g) in the form of a varnish on the surface of the prostheses [67]. In general, the results of these studies indicated a reduction in mucosal inflammation and microbial load.

In 1998, a randomized clinical study compared the effectiveness of fluconazole 50 mg (systemic) and nystatin (mouthwash), wherein fluconazole demonstrated superior results in comparison to nystatin [66]. Furthermore, the application of fluconazole has been shown to lead to a reduction in the adherence of *Candida* spp. to epithelial cells [97].

Despite the noteworthy technological advancements and innovative approaches in various fields, including dentistry, it is notable that traditional hygiene methods, particularly denture brushing, remain the most employed by patients [159,160]. However, during this period, several studies reported contradictory findings regarding the efficacy of brushing compared to chemical methods.

The superior performance of chemical methods compared to brushing in removing and killing bacteria from removable dentures was asserted in a study from 1991 [117]. Conversely, other studies indicated better results with brushing [8,10,16,25,26,38,98]. These studies underscore the importance of effective brushing accompanied by information on oral health care and awareness of daily hygiene practices.

Notably, instructions on the manual brushing of complete dentures, coupled with the use of a biofilm-disclosing agent to enhance the visualization of deposits on the denture surface, have proven effective in controlling denture biofilm and reducing mucosal inflammation [4,8,13,15,99,100].

The main objective of brushing the prosthesis is to disorganize and promote the removal of biofilm. Aiming to expand the action of toothpaste, two toothpaste formulations were developed with synthetic antimicrobial agents: chloramine-T and a fluorosurfactant (Zonyl R) [28]. Both dentifrices decreased biofilm coverage when compared with conventional dentifrice (Colgate). Dentifrice with chloramine-T was the best treatment to reduce *Streptococci mutans*, but no dentifrice influenced the microbial load of the *Candida albicans* or non-*albicans* species. Concerning dentifrices, it is important to know their abrasion capacity. When compared specifically with conventional dentifrices, was observed that specific toothpastes for dental prosthetics (Bony-plus and Dentu-creme) tend to be less abrasive than conventional ones (Colgate) [133]. Moreover, in the pursuit of enhancing brushing efficacy, studies have compared specific dental prosthesis brushes (Bitufo; Medic Denture) with conventional brushes (Colgate), yielding similar results in terms of biofilm reduction [134]. In 2006, the effectiveness of three brushes (Oral B40, a conventional toothbrush (Oral B); denture, a denture-specific brush (Condor); Johnson & Johnson, a denture-specific brush (Johnson & Johnson)) was assessed alongside a biofilm-disclosing agent in complete denture cleaning [118]. The use of the disclosing agent proved more effective in biofilm removal, irrespective of the brush that was employed. Notably, the denture-specific brush (Denture) exhibited greater efficiency than other brushes when used without the aid of a disclosing agent.

Concerning the chemical method, spanning from 1990 to 2009, numerous disinfectant solutions, both synthetic and natural, were investigated for their potential to reduce biofilm and their associated adverse effects. Notably, there was a predominant focus on effervescent solutions during this period, and few studies using sodium hypochlorite, with excipient evidence. Effervescent sanitizers underwent scrutiny in both in vitro studies [38,70,119,120,121,135] and clinical evaluations [8,35]. These studies aimed to assess their impact on the properties of prosthetic materials, their antimicrobial efficacy, and their capacity to eliminate biofilm.

Within this category, alkaline and neutral peroxides, with and without enzymes, as well as sodium perborate, were studied. Alkaline-peroxide-based sanitizers demonstrated superior efficacy against *C. albicans* when compared to those containing enzymes [119]. Alkaline peroxides without enzymes and neutral peroxides with enzymes induced changes in the color and roughness of denture reline and various acrylic resins [80,120,121]. However, the use of alkaline peroxide for 5 min [8] and sodium perborate did not demonstrate significant biofilm removal or antibiofilm action [35,38].

In this timeframe, limited studies explored the efficacy of disinfectants such as 0.2% chlorhexidine digluconate for the immersion of dentures, either alone or in conjunction with local antifungals [52], and 0.12% chlorhexidine digluconate [101]. While these studies reported satisfactory results in terms of DRS improvement and microbial load reduction, the recovery of oral flora was observed after a 15-day suspension of treatment [51,102].

Regarding sodium hypochlorite, the daily immersion of dentures in a 0.05% sodium hypochlorite solution for 10 min has been shown to effectively reduce the microbial load, particularly when associated with denture brushing [122]. Immersion in 0.5% and 1% sodium hypochlorite for 20 min daily over 180 days did not induce changes in the physical and mechanical properties of acrylic resins for denture bases polymerized by microwaves [38]. However, contrasting results were reported, showing that under similar conditions, microwave-polymerized acrylic resins exhibited color changes and decreased flexural strength [136]. Additionally, changes in color and roughness were noted in specimens composed of various materials for removable prostheses (soft and rigid reliners, and different acrylic resins) after immersion in sodium hypochlorite [120,121].

Regarding natural solutions, formulations were evaluated regarding the reduction in inflammation and microbial load. *Zataria multiflora* reduced inflammation but did not significantly reduce microbial load when compared to miconazol [78]. A propolis-based gel formulation promoted improvements in signs of inflammation and its effect was attributed to its anti-inflammatory and antifungal properties [103]. These studies demonstrated good results as treatment options for *Candida*-associated DRS or biofilm control.

The combination of mechanical brushing followed by immersion in a chemical agent, such as sodium hypochlorite and an effervescent tablet, was also reported in clinical and in vitro studies as an effective method for biofilm control [26,37,38] and certainly guided the studies carried out later.

In addition to the presented strategies, surface modification was introduced as an innovative approach to control biofilm growth on abiotic surfaces, but these results were contradictory. A study from 2000 [55] shows that glazing the inner surfaces of the dentures reduced bacterial load, while another study [56] demonstrated that glazing presented a larger tendency toward biofilm accumulation than polishing the surface. The incorporation of nystatin, amphotericin B, and chlorhexidine into the thin-film PMMA polymer reduced biofilm formation, with chlorhexidine achieving a remarkable 98% reduction [104].

From 2010 to 2023, the scientific literature witnessed a notable surge in epidemiological clinical studies, in vitro investigations, and, predominantly, randomized controlled clinical studies.

Once again, the utilization of antifungal agents for the treatment of oral mucosa, such as ketoconazole, fluconazole, and tolerable tissue plasma (TTP), was compared with nystatin (control), and 0.12% chlorhexidine mouthwash was assessed. The results indicated that ketoconazole and fluconazole exhibited satisfactory outcomes in reducing inflammation. TTP demonstrated a reduction in erythema; however, its potential benefits in addressing complaints and microbial load were not evident [68,70,81]. The topical application of 2% ketoconazole in orabase demonstrated comparable efficacy to the administration of ketoconazole (200 mg/day) in the clinical treatment of DRS, effectively reducing microbial counts [68]. Even so, among the suggestions of treatment for mucosal inflammation, it was proposed that the patient should refrain from using their denture for two weeks alongside the topical application of antifungal medication, and, after the infection subsides, a new denture should be created with proper vertical dimensions [92]. However, many patients experience inflammation and/or infections when using new dentures, which stretch and are functionally not recommended for replacement.

To facilitate the patient’s hygiene process and achieve favorable outcomes in terms of cleaning and antimicrobial efficacy, toothpaste containing synthetic or natural microbial agents has been proposed. Several in vitro studies have assessed the abrasive effects of toothpaste [135], while both in vitro and clinical studies have investigated its biofilm removal capacity and antimicrobial action [105,135]. Noteworthy agents incorporated into toothpaste formulations include *Ricinus communis* [135], resinous oils such as *Copaifera officinalis* and *Pinus strobus*, and essential oils like *Eucalyptus citriodora* and *Melaleuca alternifolia* [137].

Experimental dentifrices were commonly compared with commercial dentifrices for natural teeth or those specifically designed for dentures. The findings from in vitro and clinical studies were promising regarding the abrasiveness, biofilm removal capacity, and antimicrobial action of experimental toothpaste compared to commercial toothpaste. In comparison to conventional dentifrices, those specifically designed for dentures induced greater mass loss and less roughness on the acrylic resin [133]. Additionally, a study evaluated the combination of artificial saliva (Oral Balance) with a commercial dentifrice (Corega Brite) [138], demonstrating superior antimicrobial action compared to the exclusive use of toothpaste.

The evaluation of brushing with low-pressure oral irrigation (Waterpik) for maintaining oral hygiene in individuals with implant-retained prostheses (overdentures) revealed mixed outcomes. While the system did not demonstrate effectiveness in reducing microbial counts based on overdentures [106], the use of oral irrigation (Waterpik) proved effective in diminishing the modified plaque index, gingival index, probing depth, and bleeding index on probing. Additionally, it contributed to a high level of user satisfaction with overdentures [107].

In this timeframe, mechanical strategies, including ultrasound application [22,24,29,108], as well as physical interventions such as photodynamic therapy employing Photogem and microwave irradiation [92,109], were investigated. The findings indicated that these techniques were efficient in reducing microbial load. However, a cautious evaluation is essential before considering daily patient application.

The chemical cleaning methods under investigation included effervescent tablets and gels containing alkaline peroxide with enzymes (Corega Tabs, Polident, Toughdent, Ortoform, Bonyplus) or without enzymes (Nitradine), sodium hypochlorite (in various concentrations), surfactants, microbial agents, chlorhexidine, and natural agents such as copaiba oil, tea tree oil, *Ricinus communis* solution, and propolis [9,22,23,24,38,40,80,82,89,92,105,108,110,111,123,124,125,135,139,140,161]. The findings from studies published between 2010 and 2023 indicate that sodium hypochlorite stands out as the most effective and cost-efficient solution for biofilm control. However, concentrations exceeding 0.5% have been observed to promote alterations in the physical and mechanical properties of the acrylic resin used for denture bases and teeth, along with corrosive effects on metal alloy components [135,141]. Regarding the impact on soft liners, it was observed that the 1% sodium hypochlorite solution resulted in more pronounced changes in color, roughness, and hardness compared to the 2% *Ricinus communis* solution [135].

To mitigate the adverse effects of hypochlorite and validate its antimicrobial potential, both in vitro and clinical studies have assessed the use of hypochlorite at lower concentrations. The results generally indicate that a sodium hypochlorite solution, when used at concentrations below 0.2% for daily immersions of 20 min, exhibits clinical efficacy in biofilm removal, antimicrobial action, and improvements in inflammation, all without substantial alterations to the properties of clinically acceptable materials. It is noteworthy that most of these studies consistently applied the solution in conjunction with brushing. Sodium hypochlorite has emerged as one of the most scrutinized disinfectant solutions when used in tandem with brushing [23,24,88,112,123]. Nonetheless, there is a study that presents contradictory findings. In this research, peroxide solutions were deemed more effective in mitigating inflammation and reducing the microbial load of *S. mutans* in comparison to sodium hypochlorite [44].

The sole contraindication identified for the use of sodium hypochlorite is its inadvisability for individuals with allergies [162]. Additionally, for removable partial dentures, caution is warranted due to the oxidizing effect of sodium hypochlorite, which may have adverse effects on the metal components [141]. In such cases, alternatives are recommended, including effervescent tablets, cetylpyridinium chloride, peracetic acid, and chlorhexidine [48,126,127,136,139,142,143]. Nevertheless, it is essential to acknowledge that these alternative solutions also have their limitations. While studies suggest that effervescent tablets containing alkaline peroxide can reduce the microbial load of biofilm [23,24,37,40,110,153], the immersion-time protocols exhibited variability, ranging from 3 min (Polident) and 5 min (Corega Tabs) to 30 min (Corega Tabs), 8 h (Ortoform), and overnight (Corega Tabs Antibacteriano). In one study [23], better effectiveness was achieved with Corega Tabs when applied for 30 min compared to 5 min. However, there is a discordance in results across studies concerning the efficacy of reducing *C. albicans* [23,24,40]; the observed variations in efficacy can be attributed to differences in the compared protocols, duration of use, and analysis methodologies. An overnight immersion in alkaline peroxide, simulating a year and a half of use, did not adversely affect the flexural strength of acrylic resin but resulted in noticeable color alterations [140]. Combining mouthwash with 0.12% chlorhexidine and the immersion of prostheses in effervescent tablets with alkaline peroxide did not yield significant differences in the reduction in colony-forming units (CFU) [110]. Regarding mouthwashes (Cepacol, Plax, and Periogard), the results for their use appear more promising regarding protection against bacteria, particularly *S. mutans*, with chlorhexidine demonstrating the most favorable outcome [105].

Physical hygiene methods, including photodynamic therapy and microwave irradiation, have been evaluated [92,109], showing that photodynamic therapy demonstrates efficacy in inactivating microorganisms, particularly *Candida* spp. [109], and in reducing inflammation in patients with DRS associated with *Candida* spp. when compared to the topical effect of miconazole [71]. Additionally, another study illustrated [74] the effectiveness of miconazole and photodynamic therapy (PDT) in reducing *Candida* burden and enhancing the quality of life related to oral health in patients with denture stomatitis and type 2 diabetes mellitus, suggesting this a promising treatment option for individuals with these conditions who wear implant-retained dentures. In a randomized clinical study comparing the effectiveness of photodynamic therapy with nystatin on the prevalence and CFU of *Candida* spp. in patients with DRS, a substantial effect size was observed on the palate in the nystatin group (1.79), while the effect size for the photodynamic therapy group was moderate (0.63). Moreover, a very large effect size was noted for prostheses in both groups (nystatin: 3.01; photodynamic therapy: 1.58) [72].

In the study by Ribeiro et al. (2022) [144] assessing the quality of life and satisfaction of individuals using complete dentures concerning hygiene protocols, four protocols were examined: brushing associated with immersion in 0.25% sodium hypochlorite, 0.15% triclosan, effervescent tablets (BonyfAG), or effervescent tablets (BonyfAG) combined with palatal brushing. The findings revealed an enhancement in overall patient satisfaction, satisfaction with the maxillary prosthesis, comfort with the maxillary prosthesis, and retention of the maxillary prosthesis [144]. Conversely, another study explored the connection between the hygiene of complete dentures and the quality of life related to oral health in completely edentulous individuals [128]. In this study, involving 80 participants, no discernible relationship was identified between quality of life and prosthetic hygiene.

In the quest to develop effective and affordable products, a study has explored microbial surfactants derived from bacteria like *Acinetobacter calcoaceticus*, *Rhodococcus erythropolis*, and *Nocardia vaccinii*. These surfactants exhibited the capacity to reduce the adhesion of various microorganisms, including *Proteus vulgaris*, *Staphylococcus aureus*, *Pseudomonas aeruginosa*, *Enterobacter cloacae*, *Escherichia coli*, and *C. albicans*, on the surface of dental prostheses [111]. Furthermore, 0.75% tea tree essential oils and 10% copaiba oil demonstrated the ability to inhibit the adhesion of *C. albicans* strains to thermopolymerizable resin when compared to 1% sodium hypochlorite [82]. In the domain of natural products used for managing DRS, a gel coupled with herbal grapefruit seed extract (Citrosept) resulted in a 75% remission of grade 1 and 2 inflammation. However, there was no discernible improvement in the inflammation of the oral mucosa in patients with type 3 inflammation [85].

Additionally, in the domain of natural products, oils such as tea tree and lemon grass have demonstrated efficacy in controlling *C. albicans* biofilm on denture base resin and induced acceptable alterations in the surface wear of the resin [86]. In another study, the effectiveness of mouthwash and a mouth spray containing *Cinnamomum zeylanicum Blume* essential oil, in comparison to the control group with nystatin, was assessed [88]. The authors demonstrated that both treatments could reduce the microbial load of *Candida* spp. on the palatal mucosa and prostheses, leading to symptom improvement and clinical regression of the lesion. Therefore, cinnamon essential oil holds promise as a promising alternative treatment for oral candidiasis associated with the use of dental prostheses. Furthermore, the use of a chitosan solution (5 mg/mL) was also evaluated, which exhibited a promising antimicrobial effect when immersing acrylic resin and Co-Cr alloys [139].

Several studies in this decade have explored innovative approaches to enhancing dental prosthesis materials. These include the application of varnish (Sterngold), glazing adhesive (Biscover LV and surface coat), titanium dioxide (TiO_2_) coatings using the atomic layer deposition (ALD) technique, and the incorporation of antifungal agents such as 5% miconazole, characterized nickel, and nickel oxide nanoparticles (NiCl_2_, NiNPs, and NiONPs), derivatives of the compound 1,4-diazabicyclo [2.2.2]octane (DABCO), Yamani henna powder, and silver (AgNPs) and titanium oxide (TiO_2_ NPs) nanoparticles, into dental prosthesis manufacturing materials [53,57,61,69,79,84,87,90,113].

The application of varnish did not lead to a reduction in biofilm adhesion and caused deterioration of the material’s surface. On the other hand, the glazing agent increase the smoothness of the surface, but it lacked stability when exposed to sodium hypochlorite for disinfection. Coating with TiO_2_ significantly improved wear resistance and reduced *C. albicans* biofilm formation compared to uncoated PMMA. The incorporation of miconazole into the acrylic resin prevented the formation of hyphae by *C. albicans*; however, it caused damage to oral epithelial cells and triggered a strong epithelial pro-inflammatory response. The incorporation of antimicrobial agents into the material facilitated a reduction in microbial growth [53,57,61,69,79,84,87,90,113].

Most in vitro studies during this timeframe centered on integrating natural or synthetic nanomaterials with potential antimicrobial properties into polymethyl methacrylate (PMMA) specimens. The exploration of natural substances included herbal remedies like *Equisetum giganteum* and *Punica granatum* [83], along with essential oils such as tea tree and lemon grass [86]. Moreover, investigations delved into the effectiveness of *Cinnamomum zeylanicum Blume* against *C. albicans*.

In the study by Almeida et al. (2018) [83], the antimicrobial activity of herbal medicines exhibited noteworthy effectiveness when integrated with PMMA. This was evidenced by a decrease in metabolic activity, modifications in biofilm microarchitecture, and a more confined distribution of viable and non-viable fungal cells.

Concerning the synthetic nanomaterials that were employed, notable examples include titanium dioxide nanoparticles (TiO_2_NPs) [87,90], silver nanoparticles (AgNPs) [90,114], and nanostructured silver vanadate adorned with silver nanoparticles (AgVO_3_ and β-AgVO_3_) [145]. The integration of TiO_2_NPs and AgNPs into PMMA at concentrations of 0.5% or 1% demonstrated a reduction in *C. albicans* biofilm bioactivity and biomass [90]. The inclusion of TiO_2_NPs in PMMA resulted in diminished *C. albicans* biofilm formation, the heightened hydrophobicity of PMMA, and no compromise to the material’s flexural strength [87]. AgVO_3_, when incorporated at a 10% concentration, exhibited antimicrobial activity against *S. mutans* within a multispecies biofilm also composed of *C. albicans* and *C. glabrata* [145].

In the context of complete denture relining materials, AgVO_3_ demonstrated antimicrobial efficacy against *Enterococcus faecalis*, *P. aeruginosa*, and *C. albicans*. Importantly, this was achieved without compromising the material’s roughness and hardness, while simultaneously enhancing the adhesion between the reliner and the denture’s acrylic resin [146]. Furthermore, it has been established that varying concentrations of β-AgVO_3_ exhibit antibacterial activity; however, they may adversely impact the roughness, hardness, impact resistance, and flexion of the acrylic resin [147].

The combination of AgNPs with Corega adhesive exhibited remarkable antimicrobial activity against *C. albicans* biofilms for up to 12 h, suggesting potential preventive or therapeutic effects against denture stomatitis [114].

In an in vivo, ex vivo, and in vitro study [81], mucoadhesive nanoparticles containing fluconazole and coated with chitosan were locally applied to the oral mucosa of rabbits infected with *C. albicans*. The study revealed antimicrobial efficacy and favorable outcomes, not only in reducing the overall required dosage and minimizing the side effects but also in eliminating potential drug interactions encountered during systemic fluconazole therapy. Importantly, the formulation demonstrated no cytotoxic effects at the tested concentrations through in vitro evaluation.

The inclusion of 5% miconazole in the MAA-UDMA resin led to reduced damage and scaling of epithelial cells [69]. In vitro assessments of resin compositions incorporating 5% miconazole, characterized nickel, and nickel oxide nanoparticles (NiCl_2_, NiNPs, and NiONPs), and derivatives of the compound DABCO and Yamani henna powder, yielded favorable results in microbial reduction [61,69,79,84]. NiCl_2_ nanoparticles and NiNPs exhibited superior performance in inhibiting biofilm formation and microbial growth compared to NiONPs [79]. While the compound DABCO demonstrated high cytotoxicity for oral cavity cells, its derivatives DC11MAF and C2DC11MAF exhibited low cytotoxicity and the ability to prevent *C. albicans* biofilm formation [84]. Additionally, derivatives DC11MAF and C2DC11MAF, along with Yamani henna powder and 5% miconazole, effectively reduced *C. albicans* counts [61,69,84].

The existing literature underscores the importance of concurrently assessing the clinical efficacy of treatment and maintenance therapy for DRS alongside ongoing management of the patient’s existing chronic conditions [75,144], highlighting the significance of multidisciplinary investigations. This holistic approach towards patients with concurrent chronic diseases who are positive for DRS was executed within distinct cohorts manifesting gastrointestinal pathology (predominantly chronic gastritis, Group I), type 2 diabetes (Group II), and cardiovascular system pathology (primarily coronary heart disease; Group III) [75]. Prosthesis adaptation and disease-specific treatments, encompassing Altan and Tantum Verde lollipops (Group I), Tiotriazolina and Lizak (Group II), Biol, coenzyme Q10, and Lisobakt DUO (Group III), were administered to participants. Following treatment, individuals underwent a structured maintenance therapy. The outcomes revealed a cessation of oral lesions linked to DRS and the prevention of DRS recurrence over a year in 78% of the patients. An assessment involving thirty-three individuals was conducted, examining their status before and after a 10-day hygiene protocol treatment. This treatment regimen comprised palatal brushing with a soft brush using water, denture brushing with a denture-specific brush and mild soap, and denture immersion in a 0.25% sodium hypochlorite solution for 20 min. The variables under scrutiny encompassed DRS remission, biofilm elimination, microbial load, salivary MUC 1, cytokine levels, and arterial pressure. The outcomes revealed that the implemented hygiene protocol effectively diminished the inflammation associated with DRS. Furthermore, an improvement in local inflammatory factors and a reduction in systolic arterial pressure were observed among the participants [144].

Regarding the influence of adhesives on DRS, a recent randomized clinical study investigated the microbiological efficacy of effervescent tablets, specifically Corega Tabs, in prosthetic hygiene when adhesives were employed. The experimental group utilizing adhesive and cleaning dental prostheses with Corega Tabs exhibited a significant reduction in microbial load. Consequently, the study recommends the use of Corega Tabs for prosthetic hygiene in cases involving adhesive use [115].

In alignment with this research focus on prosthetic adhesives, another study explored the variability in the biofilm and the efficacy of hygiene methods based on different adhesive presentations, such as cream, strips, and powder [148]. The findings revealed a higher microbial load of *C. albicans* with strip or powder adhesive, an elevated load of *S. aureus* with strip adhesive, and an increased *P. aeruginosa* load with cream adhesive. When assessing the use of various hygiene protocols to remove adhesive adhered to the prosthesis (brushing with distilled water, Protex soap, Colgate dentifrice, immersion in Corega Tabs, and immersion in Corega Tabs followed by brushing with the solution itself), the most effective method was determined to be brushing with Protex soap.

In addition to the development of products and methods that aim to reduce the microbial load and prevent DRS, technological development also provides new materials for application in the manufacture of prostheses, such as additive manufacturing (printed) and subtractive manufacturing resins. Aiming to evaluate the behavior of these materials in the face of biofilm formation, and their physical–mechanical behavior when exposed to different environmental factors and hygiene protocols, some in vitro studies have been carried out [116,129,130,131,132,149].

In this context, an experimental study observed that the use of brushing combined with 0.25% sodium hypochlorite for 20 min effectively reduced the microbial load of multispecies biofilm. However, they noted that the printed resins experienced alterations in their physical–mechanical properties when evaluated over a simulated period of 5 years [149]. Consequently, caution is warranted when recommending the prolonged use of these resins. In another study, *C. albicans* adhesion was investigated and it was found that the propensity for fungal biofilm attachment varies among different resin types, with a higher tendency observed for 3D-printed resin surfaces [129]. Studies that evaluated *Candida albicans* formation in different types of resins, such as thermopolymerized PMMA/conventional resin, and milled and printed resins, demonstrated greater adhesion, higher counts of colony-forming units, and the formation of *Candida albicans* hyphae in printed resins compared to conventional and milled resins [129,131,132]. Furthermore, as printed resins present physical–mechanical changes compared to conventional resins, it is recommended to use printed resins for the manufacture of denture bases for temporary rehabilitative treatments that are restricted to less than 3 years [149]. Innovatively, a 3D-printed denture base resin material modified with mesoporous silica nanocarriers loaded with silver in varying proportions (0.0–2.0 wt%) (Ag/MSN) was developed to enhance the mechanical and antimicrobial properties. While some properties exhibited changes, the technique demonstrated significant efficacy in enhancing antimicrobial activity against *C. albicans* [130].

Several removal methods, including microwave irradiation, mechanical brushing, and Polident overnight tablets, demonstrated comparable efficacy in eliminating *C. albicans* from all types of polymethyl methacrylate (PMMA), including 3D-printed resin. Conversely, glutaraldehyde exhibited less effectiveness. Moreover, it was established that 2% chlorhexidine gluconate and 0.5% or 1% sodium hypochlorite resulted in zero colony growth for both *C. albicans* and *S. mutans*. In terms of material groups, 3D-processed discs exhibited an increase in CFU, followed by the conventional and computer-aided design and computer-aided manufacturing (CAD/CAM)-milled group. Notably, 3D-printed discs demonstrated the highest surface roughness [131].

## 3. Materials and Methods

The scoping review was conducted following the PRISMA-ScR guidelines that ensure the transparency and completeness of the review process [163].

### 3.1. Search Strategy

A bibliographic search was carried out using PubMed, Embase, Web of Science, and Scopus, without date filters. Mesh terms, synonyms, and free terms referring to the population, intervention, and comparison were combined with Boolean operators “OR” and “AND”. The search strategy was initially developed for PubMed and later adapted for other databases using specific syntax rules. The Mesh Terms (“Biofilm”, “Bacterial adhesion”, “Candidiasis, Oral”, “Stomatitis”, “Stomatitis, Denture”, “Mucositis”, “Colony-Forming Units Assay”, “Acrylic Resins”, “Metal Ceramic Alloys”, “Denture Liners”, “Denture Bases”, “Tissue Adhesives”, “Mouth Mucosa”, “Tooth”, “Mouth, Edentulous”, “Infection Control”, “Therapeutics”, “Preventive Dentistry”, “Health Education Dental”, “Denture Cleanser”, “Oral Hygiene”, “Antifungal Agents”). Through the application of search keys within the corresponding databases, articles spanning the period from 1969 to 2023 were identified.

### 3.2. Eligibility Criteria

For this review, we considered articles referring to conventional and any new technology applied to evaluate the formation and quantification of biofilm on abiotic (acrylic resin, reliner, adhesive, and metal alloys) and biotic surfaces (teeth and epithelial mucosa) via analysis of biofilm formation, microbial load, colony-forming units (CFU), biofilm removal, and cell viability. Furthermore, the most recommended methods and products for the hygiene of the surfaces were evaluated.

Randomized controlled trials, nonrandomized comparative studies, observational studies, and cross-sectional studies, and in situ and in vitro studies for the analysis were included in this study. Case reports, literature reviews, systematic reviews, letters to the editor, conference abstracts and expert notes, and studies related to periodontal disease, infections or inflammations caused by systemic diseases, and non-involvement with complete dentures or partial removable dentures were excluded. Studies conducted worldwide were considered, considering partial and complete edentulous patients who are users of prostheses. No language restrictions were applied.

### 3.3. Study Selection

Eligible citations were retrieved, and the data were exported to EndNote before being uploaded to Rayyan QCRI. Mechanical evaluation and subsequent human decisions were employed to eliminate duplicate entries. The selection process comprised three phases: (1) a title and abstract screening conducted by nine reviewers, (2) a full-text screening of the selected articles performed by the same reviewers, and (3) a final screening during the data extraction phase to exclude articles with identical outcome measures or data that could not be extracted.

## 4. Discussion and Conclusions

Through the bibliographical research carried out in this study, we found that in the period from 1969 to 1989, few studies were found on the treatment and prevention of infections in the oral mucosa or controlling biofilm related to the use of prosthetic devices and, among the published studies, most are clinical studies. In the first decade of the 21st century, an important change in this scenario was noted, with in vitro and in vivo studies examining various methods for DRS treatment, controlling biofilms on the surfaces of teeth, prostheses, and implants, and treating infections in the palatal mucosa. Investigations related to the oral hygiene of denture wearers were also carried out and the results helped to understand how cleaning methods are used and what their relationship is with diseases associated with biofilm accumulation. Although one study reported the absence of a relationship between the presence of DRS and the frequency or method of cleaning dentures [11], different studies showed a reduction in the degree of inflammation after the institution of protocol hygiene or the replacement of dentures [4,13,25]. The approach of replacing dentures [5] was later corroborated by the finding that older dentures had a dirtier appearance [12] and that the type of acrylic resin used in their fabrication, as well as the polishing process, have a substantial influence on microbial adhesion [7].

From 2010 to 2023, a notable surge in research activity occurred, with randomized controlled clinical studies, cross-sectional clinical studies, and in vitro studies. The clinical investigations aimed to assess various biofilm control strategies, ranging from the home use of biofilm-revealing solutions by patients to the evaluation of dentifrices and disinfectant solutions for dentures, incorporating synthetic and natural active ingredients [116]. These interventions were often combined with ultrasound and palate brushing, or conducted with palate brushing alone [22,32,33,80,89,105,106].

Over this period, a notable evolution in the research landscape is evident, particularly characterized by a substantial increase in controlled clinical studies, contributing a high level of evidence to the area [18,24,27,40,44,57,68,70,71,72,74,75,85,89,92,110,112,115]. Employing crossover or parallel group models, these studies explored several methods, with particular emphasis on the combination of brushing and disinfection using solutions and indicated anti-biofilm effectiveness, as well as the remission of inflammation results that can be considered clinically relevant. However, the uncontrolled and randomized studies found in this review have played a fundamental role in evaluating the physical, mechanical, and chemical properties of dental materials, evaluating their biocompatibility and investigating the effectiveness of agents. Although these studies present limitations regarding the power of scientific evidence in clinical decision-making, it is important to recognize that these studies generate questions about the topic, encouraging clinical trials.

Epidemiological investigations have consistently identified the predominant demographic of denture users as the elderly [9,14,18,21]. This population often exhibits suboptimal oral hygiene practices, as evidenced by the presence of biofilm [14,29] or quantifiable microorganisms in the oral cavity [18]. Contributing factors to inadequate hygiene include motor difficulties in performing effective brushing techniques, improper prosthesis use, insufficient information, or caregiver limitations in prosthesis handling. The quality of instructions provided for prosthesis cleaning emerges as a crucial determinant for achieving satisfactory hygiene [9,14,18,29,50,113]. During this timeframe, DRS emerged as the most prevalent oral disease among complete denture users. Its occurrence was attributed to inefficient brushing techniques, prolonged denture use, and the nocturnal use of dentures [9,14,49]. These findings underscore the imperative for effective hygiene guidelines and methods to manage denture-related oral health concerns.

Surveys investigating denture hygiene practices consistently highlight brushing as the most prevalent method among denture users, typically involving the use of a toothbrush paired with water, soap, or toothpaste [9,14]. Chemical agents are reported with lower frequency, with effervescent sanitizing tablets containing alkaline peroxide [18] and sodium bicarbonate [47,49] being the most used by patients.

Recent clinical studies suggest that the combination of brushing with sodium hypochlorite stands out as a highly effective method for biofilm control and the prevention of DRS. Notably, this approach exhibits safety concerning its impact on the resinous materials used in prosthesis fabrication [88,116,129,144]. These findings have clinical significance, given the simplicity of the brushing method and the ready availability and affordability of sodium hypochlorite. While certain effervescent tablets have shown favorable results, their accessibility remains restricted for many patients.

Furthermore, we found that many techniques for altering materials or promoting surface modification with the incorporation of synthetic or natural agents, as well as proposals for disinfectant solutions for immersion or dentifrice based on natural products, have been found [50,90,111,113,114,120,143]. Despite these techniques and agents presenting encouraging results due their potential antimicrobial activity and acceptable effects on material properties, they are not yet a reality for application, as they are not commercialized. It is important to highlight that no information was found regarding safety recommendations concerning the use of nanoparticle-based strategies in DRS control or treatment. The authors relate the biocompatibility of the chosen particles. Thus, there are limitations in the discussion of these aspects and it is still necessary to evaluate the association between nanoparticle-based strategies and their mechanical, biological, and aesthetic properties. These notes reinforce the need for partnerships between the scientific community and companies, which must be strongly established so that the scientific knowledge that is produced can be applied to the population with security.

Regarding clinical studies, studying the impacts of inflammation and oral microbiota on systemic diseases and the health of the oral cavity is essential for treating the individual. Considering that most of the target population of the studies are elderly and have several comorbidities, controlling all factors that may influence the general health and quality of life of these individuals can contribute to increasing their life expectancy, as well as reducing investments in medicines and the healthcare system. Another noted issue is the importance of education programs regarding the use of prostheses and the prevention of oral diseases related to dental elements and soft tissues. Despite the technological advances, epidemiological studies still highlight the precariousness of information and the lack of knowledge among patients.

Therefore, considering the findings, different strategies, materials, and products are involved with DRS management. Although promising results have been found, many of the proposed methods have not been made available for clinical use. Among those that are commercially available, the antifungals have limitations related to their side effects and recurrence of the disease after the period of use, and this occurs with chlorhexidine. Thus, the combination of brushing and immersion in sodium hypochlorite or alkaline peroxide seems to be the most accessible method, with the possibility of efficient clinical application, despite the limitations related to its synthetic characterization, its contraindicated use in metal prostheses, and the possibility of allergic reactions. Therefore, it turns out that new research is needed with innovative and sustainable materials, such as natural products, that show some antimicrobial potential, satisfactory local and systemic results, and effectiveness for different types of materials.

## Figures and Tables

**Table 1:** Studies for topical and systemic treatments using antimicrobial agents, either alone or associated with local interventions.

	Antimicrobial Agents	Presentation Form	Frequency
Antifungals	Nystatin	Mouthwash [66,88] Tablets (500,000 units each) [93] Ointment (100,000 IU—Nystaderm Mundgel) [70] Oral suspension (100,000 IU) [72]	1 mL four times a day for 21 days [66] Four times a day on the surface of the prosthesis for 6 weeks [70] 10 mL for 1 min, three times a day for 15 days [88] Three times a day for 14 days [93] Four times a day for 15 days [72]
	Amphotericin B	Tablets (10 mg) [52]	Four tablets, each taken at 4 h intervals throughout the day, for a period of 14 days [52]
	Miconazole	Varnish (55 mg/g) [67] Gel (2%) [67,71,74,96]	Gel (four times a day for 2 weeks) [67] Varnish (applied once to the mucosal surface of the prosthesis) [67] Three times a day for a month [71] Four times a day for two months [74] Three to four times a day for four weeks [96]
	Fluconazole	Capsule (50 mg) [66,96]. 2 μg mL^−1^ [97]	One capsule per day for 7 days [66] Once a day for 2 weeks [96]
	Tolerable tissue plasma (TTP)	TTP irradiation [70]	One time a week for six weeks [70]
	Ketoconazole	Orabase (2%) [68] Tablet (200 mg/day) [68]	Tablets (once a day for 14 days) [68] Orabase (twice a day for 14 days) [68]
	Chitosan	Solution (5 mg/mL) [139]	Does not contain this information
Natural agents	*Ricinus communis*	Solution (2%) [80,135,139]	Three-year simulation with 20 min daily immersion [80,135,139] One-and-a-half-year simulation with daily immersion for 8 h (overnight) [135]
	Tea tree	Essential oils (0.188%, 0.375%, 0.5%, 0.75%, 1%) [82,86]	Soak in the solutions for 1, 3, and 5 min [86]
	Copaiba	Oil (10%) [82]	Does not contain this information
	Herbal grapefruit seed extract	Citrosept Gel (1%) [85]	Three times a day for three weeks [85]
	Lemon grass	Essential oil (0.125%, 0.25%, 0.5%, 1.0%, 0.5%) [86]	Soak in the solutions for 1, 3, and 5 min [86]
	*Cinnamomum zeylanicum* Blume essential oil	Essential oil spray (0.5 mg/mL) [88]	Spray on dentures three times a day for 15 days [88]
	Propolis	Gel [103]	Four times a day for one week [103]
	Salivary polypeptides rich in histidine	Mouthrinse (histidine-rich polypeptides 3 or 4) [65] Denture soak (histidine-rich polypeptides 3 or 4) [65]	Mouthrinse twice a day for a period of 1 week [65] Overnight denture soak [65]
	*Zataria multiflora*	Gel (0.1%) [78]	Four times a day for 2 weeks [78]

**Table 2:** Mechanical, chemical, physical, and associated methods used for the prevention and/or treatment of DRS.

	Methods	Presentation Form	Frequency
Chemical	Chlorhexidine Digluconate	Solution (0.02%, 0.12%, 0.15%, 0.2%, 0.3%, 1.25%, 2%, 2.5%) [36,51,52,101,102,131], Gel (1% gel) [95] Tablet (5 mg) [52] Mouthwash 0.12% [70,122,131]	Four times a day at 4 h intervals throughout the day for 14 days [51] 15 min in solution [52] Twice a day for 60 s after removing the prostheses for 6 weeks [70] Twice a day for 1 month [95] 15 s twice a day for 14 days [102]
	* Sodium Hypochlorite	Solution (0.05%, 0.25% 0.45%, 0.5% and 1%) [23,38,44,82,120,121,122,131,135,136,140,141,144]	10 min immersion over 15 days [122] 20 min soak daily for 180 days [38] 10 min for 6 weeks [23] 12 h a day for 365 days [121] Three-year simulation with daily immersion for 20 min [135] One-and-a-half-year simulation with daily immersion for 8 h (overnight) [135] 180 immersions of 10 min each [136,141] Continuous immersion for 182 days [140] 20 min a day for 10 days [144] 0.5% (20 min) and 1% (10 min) [131]
	* Peroxide solutions (Effervescent Tablets)	Bonyplus^®^ [37] Efferdent^®^ [117] Corega Tabs^®^ [23,40,115,140] Steradent and Superdrug^®^ [120] Steradent and Polident^®^ [121] Polident^®^ [129] Corega anti-bacteria denture cleanser tablets^®^ [24]	Corega Tabs (5 min per day for 6 weeks) [23] Corega Tabs (30 min a day for 6 weeks) [23] Corega Tabs (overnight immersion for 14 days) [40] Corega Tabs (continuous immersion for 182 days) [140] Corega Tabs (6 months of use) [115] Bonyplus (5 min a day for 7 days, repeated three times) [37] Steradent and Superdrug (10 min soak cycles repeated at 0, 1, 3, 7, 14, 21, and 50 cycles) [120] Steradent and Polident (12 h of immersion per day for 365 days) [121] Polident (8 h of immersion) [129]
	Cetylpyridinium Chloride	Cepacol (0.500 mg) [136,141]	180 immersions of 10 min each [136] 10 min for each immersion, resulting in 1800 min [141]
	Microbial surfactants	Acinetobacter calcoaceticus bacteria (0.003–0.036 mg/mL) [111] Rhodococcus erythropolis bacteria (0.03–0.12 mg/mL) [111] Nocardia vaccinii bacteria (0.005–0.05 mg/mL) [111].	Does not contain this information
	Glutaraldehyde	Solution (2.5%) [129]	90 min immersion [129]
* Mechanical	* Brushing of complete dentures and mucosa	Dentifrice/brush [2,8,15,25,26,99,100] Brush [30,31,98] Biofilm-disclosing agent [8,100]	Brush twice a day for 2 min [2] Brush twice daily for 2 min for 60 days [30] Duration of 60 days [31] 2 min daily for 60 days [8] 3 times a day for 60 days [25] Daily for 6 weeks [26] Twice a day for 30 s for 6 weeks [98] 20 s [99] 2 min daily for 14 days [100]
	Toothpaste formulations	Chloramine-T (1%) [28] Fluorosurfactant (0.01%—Zonyl R) [28] Resinous oil (0.5%—*Copaifera officinalis*) [137]; Resinous oil (0.5%—*Pinus strobus*) [137]; Essential oil (0.5%—*Eucalyptus citriodora*) [137]; Essential oil (0.5%—*Melaleuca alternifolia*) [137]; Artificial saliva (Oral Balance) [138]	2 min daily for 21 days [28,138] 16.2 cycles of 3 min each [137]
	Brush	Conventional (Colgate [134] or Oral-B [118]); Denture-specific brushes (Bitufo; Medic Denture [134] or Condor or Johnson & Johnson [118])	Three daily brushings within 10 weeks [134] Three daily brushings within 6 weeks [118]
	Low-pressure oral irrigation	Waterpik [106,107]	For 2 min, 3 times a day for 28 days, with a 7-day wash-out period after 14 days [107,108]
	Ultrasound	Ultrassonic Cleaner (modelo2840 D—Odontobrás) [22] Sonorex Bandelin RK100H [24]	Once at the end of a 21-day period (15 min) [22] Daily for 5 consecutive days [24]
	* Type of dentifrice	* Water [133,138,148]; * Soap (Protex, neutral) [138,148]; * Toothpaste (Colgate) [148]; * Denture specific toothpaste (Bony-plus, Dentu-creme, Corega Brite) [133,138] * Corega Tabs solution [148]	* Once a day (2 min) for 3 weeks [138] 2-year denture cleaning simulation [133]
Physical	Photodynamic therapy	GaA1As diode laser [71,72] Suspension (50 and 100 mg/L—Photogem^®^) [110]	Twice a week, with an interval of at least 48 h between sessions, for four weeks. [71,72] 30 min (pre-irradiation solution) and 36 min during irradiation [109]
	Microwave irradiation	Microwave Sterilizer (700 W) [92,129]	Once a day (3 min) for 14 days, with a wash-out period of 30 days break 7 days [92] 3 min irradiation [129]
Associated	* Brushing and Sodium hypochlorite	0.05%, 0.2%, 0.25%, 0.5% [23,26,112,122,123,144,149]	5 and 30 min for 6 weeks [23] Soak 20 min once a week and brush daily for 6 weeks [26] Immersion for 10 min a day for 15 days [122] Brushing three times a day and soaking (20 min) for 14 days with a wash-out period on day 7 [123] Brushing for 1 min, three times a day, and immersion for 10 min, once a week for two weeks [112] Brushing the mucosa and prosthesis for 3 min, three times a day, and immersing the prosthesis for 20 min, once a day, for 10 days [144] 484 h of soaking and 60 months of brushing simulation [149]
	* Brushing and Effervescent tablets (BonyfAG)	Bonyplus [37,99] Corega anti-bacteria denture cleanser tablets^®^ [24] Corega Tabs^®^ [22] Efferdent [117]	Brushing dentures three times a day and soaking once a day (20 min) for 21 days [22] Once a day for 5 days [24] Once a day for 5 min and three times a day (2 min) for 7 days; Cycle repeated three times [37] Soaking for 5 min and brushing 20 s [99]
	Chlorhexidine Digluconate and Alkaline peroxide effervescent tablets	Mouthwash (0.12%) [110]	21-day period [110]
	Chlorhexidine Digluconate and local antifungals [54]	Miconazole (48 μg/mL) [52] Chlorhexidine Digluconate (0.2%) [52]	Does not contain this information
	Ultrasonic cleaning and Cleansing tablet	Corega anti-bacteria denture cleanser tablets^®^ [24] Corega Tabs^®^ [22] Ultrasonic Cleaner, modelo2840 D—Odontobrás [22] Sonorex Bandelin RK100H device^®^ [24]	Immersion once a day (20 min) in an effervescent tablet and brushing three times a day for 21 days. At the end of the 21 days, 15 min of ultrasonic cleaning [22] Overnight for 5 consecutive days [24]
	* Denture Cleanser Associated with Microwave Disinfection and Brushing	Microwave steam sterilizer (700 W) [92] Ortoform [92]	Brushing three times a day and once a day microwave irradiation (3 min) for 14 days with a wash-out period on day 7 [92] Brushing three times a day, once a day microwave irradiation (3 min), and overnight soaking in enzymatic cleaner (8 h) for 14 days with a wash-out period on day 7 [92]
	Relining of prostheses and subsequent replacement	Associated with antifungals [93,152] Associated with surgical removal of hyperplastic tissue [64]	Two weeks for antifungal treatment and a pause from using the prosthesis [153]

* The highlighted strategies are the most commonly used for removable dentures at present.

**Table 3:** Innovation in prosthetic materials for prevention and/or treatment.

	Presentation Form	Frequency
Coating for the surface of the prosthesis	Glaze (Biscover^®^ LV) [53] Glaze (Surface Coat^®^) [53] Glaze (Perma Cure System) [54] Glaze (Permalink^®^) [55] Varnish (Sterngold) [57]	Applied once to rough and smooth surfaces [53] Applied once to fitting denture surface [54,55] Applied once to the tissue conditioner [57]
Incorporation of the agents in acrylic resin	Nystatin [104] Amphotericin B [104] Chlorhexidine [104] Miconazole (5%) [69] Titanium dioxide (TiO 2 NPs) nanoparticles (0.5% and 1%) [87,90] Nickel and nickel oxide nanoparticles (NiCl2, NiNPs, and NiONPs—50, 100 and 200 μg/mL) [79] Yamani henna powder (1%, 2.5%, 5%, 7.5%, 10%) [61] Silver (AgNPs—0.5% and 1%) [90,114] Nanostructured silver vanadate adorned with silver nanoparticles (AgVO3 and β- AgVO3—2.5%, 5%, 10%) [145] Incorporation of derivatives of the compound DABCO (DC11MAF and C2DC11MAF—1, 2, 4 wt%) [84] Mesoporous silica nanocarriers loaded with silver in varying proportions (0.0–2.0 wt%) (Ag/MSN) to 3D-printed denture base resin material [130]	Applied once to thin-film PMMA polymer [87,104] Atomic layer deposition (ALD) technique [87] Applied once to MAA-UDMA resin [69] Applied once to 3D-printed denture base resin material [61] Added to acrylic resin powder [90] Added to heat-cured acrylic [61] Adhesive applied to heat-cured acrylic resin [114] Added to heat-cured resin powder [145] Conjugation with methacrylate monomers [84]
Fluconazole and Chitosan	Adhesives with chitosan [60] Buccal mucoadhesive nanoparticle containing fluconazole coated with chitosan [81]	Heat-polymerized acrylic resin [60] Heat-polymerized acrylic resin disks [81]
Denture adhesive	Equisetum giganteum [83] Punica granatum [83]	Applied to heat-cured acrylic resin specimens [83]
Amphotericin B	Patches (2%) [76]	Three times a day for a maximum of 2 months [76]

## Data Availability

No new data were created or analyzed in this study. Data sharing does not apply to this article.
